# Role of visual function and performance of activities of daily living in cognitive function in patients with mild cognitive impairment: a cross-sectional study

**DOI:** 10.3389/fnagi.2025.1505815

**Published:** 2025-02-05

**Authors:** Chunhui Zhou, Ganfeng Yang, Marc Theeboom, Hua Yang, Ruiting Zhu, Zijian Zhou, Dong Zhu

**Affiliations:** ^1^School of Wushu, Shanghai University of Sport, Shanghai, China; ^2^Physical Education Institute, Soochow University, Suzhou, Jiangsu, China; ^3^Department of Exercise and Sport Science, Free University of Brussels, Brussels, Belgium

**Keywords:** Alzheimer's disease, mild cognitive impairment, cognitive function, visual function, activities of daily living

## Abstract

**Background:**

Mild cognitive impairment (MCI) is a precursor to dementia, with many patients showing early decline in activities of daily living (ADLs). However, the role of visual impairment in this process is underexplored despite evidence suggesting it may accelerate cognitive decline. Current research does not understand how visual dysfunction affects cognitive abilities and how ADLs might moderate this relationship. This gap is crucial because early interventions targeting visual impairments could potentially delay progression to dementia, offering new avenues for supporting MCI patients.

**Objective:**

This study investigates the relationship between visual function and cognitive abilities in patients with MCI. Specifically, it seeks to determine how different aspects of visual function, such as visual field indices and visual acuity, correlate with various cognitive domains measured by standardized assessments. Additionally, the study aims to examine the role of ADLs as a potential moderating factor in this relationship. By analyzing how ADL performance influences the strength and direction of the association between visual impairments and cognitive function, this research intends to identify key areas where visual deficits may contribute to cognitive decline in MCI.

**Methods:**

This is a cross-sectional study. Two hundred and seventy four elderly patients were diagnosed with MCI from various communities in Taicang City, Jiangsu Province, China. Most participants were women (68.6%), and the average age was 69 years. Notable comorbidities included hypertension (41.6%) and diabetes (33.2%), indicating a higher burden of health conditions than typical older adult populations. Visual function, Montreal Cognitive Assessment (MoCA), and the ADLs were measured. Pearson's correlation coefficients were used to examine the unadjusted associations between visual function measures and cognitive performance. Multivariable linear regression models were employed to further explore these relationships and to assess the moderating role of ADLs.

**Results:**

Significant positive correlations were found between visual function and cognitive performance, particularly with Visual Function Index (VFI) showing a strong initial correlation with the total MoCA score (*r* = 0.61, *p* < 0.001), which was attenuated after adjusting for ADL (β = 0.06, *p* = 0.23). Maximum Visual Field (Max VF) was positively correlated with language skills (*r* = 0.13, *p* < 0.05). The negative correlation of Minimum Visual Field (Min VF) with cognitive performance became positive after ADL adjustment (β = 0.12, *p* = 0.04).

**Conclusions:**

These findings suggest that visual function plays a crucial role in the cognitive and functional abilities of patients with MCI. Early interventions targeting visual impairments may help mitigate cognitive decline and improve the quality of life for these patients.

## 1 Background

MCI is a pivotal phase in the spectrum of cognitive aging and represents an intermediate stage between cognitive changes typical of normal aging and those indicative of dementia, notably Alzheimer's disease (AD), with approximately 50% of individuals progressing to AD within 4–5 years (Gauthier et al., 2006). Given this uncertain trajectory, early identification, understanding of risk factors, and intervention strategies are critical to potentially delay or prevent further cognitive decline and functional impairment.

ADLs encompass a range of tasks necessary for independent living, including basic activities, such as eating and dressing (BADLs), and complex instrumental activities such, as managing finances and transportation (IADLs) (Fuentes et al., 2020). Impairments in basic ADLs typically emerge in later stages of cognitive decline; as such, deficits in IADLs may present earlier and are closely linked to cognitive abilities, such as memory, attention, and executive function (Liu-Seifert et al., 2015). Understanding the relationship between cognitive function and ADL performance is essential for assessing functional independence and designing interventions to support individuals with cognitive impairment.

Among the factors influencing ADL performance, sensory impairments—such as vision and hearing loss—are prevalent in aging populations and have been shown to accelerate cognitive decline (Heyl and Wahl, 2012). The common-cause hypothesis posits that shared underlying factors influence sensory and cognitive functions, leading to strong covariability between the two domains (Lindenberger and Baltes, 1994). The information–degradation hypothesis suggests that sensory degradation may tax cognitive resources, thereby affecting cognitive processing abilities (Schneider and Pichora-Fuller, 2000). Research highlights the complex interplay among sensory function, cognitive function, and daily function in older adults. Therefore, understanding the effect of sensory impairments on cognitive function and everyday functioning is crucial to develop targeted interventions to mitigate cognitive decline in older adults.

Among sensory impairments, visual impairment holds particular significance because of its pervasive effect on cognitive function and daily functioning in older adults. Older adults with visual impairment are more likely to report subjective cognitive complaints and experience limitations in ADLs compared with those without visual impairment (Brown et al., 2014; Lee et al., 2019). Moreover, visual disturbances, such as impaired visual acuity, contrast sensitivity, and spatial perception, are common in individuals with AD and may manifest as early symptoms of the disease (Cormack et al., 2000; Armstrong, 1996). However, despite the growing recognition of the effect of visual impairment on cognitive function, a notable gap exists in understanding such relationship in individuals with MCI. Addressing this gap is critical for several reasons. First, elucidating the interplay among visual function, cognitive function, and ADL performance in individuals with MCI may provide valuable insights into the mechanisms underlying cognitive decline in this population. Second, understanding how visual impairment influences cognitive function and everyday functioning in MCI may inform the development of tailored interventions and support strategies to address the unique needs of this population. Finally, by identifying early markers of cognitive decline in MCI, clinicians and researchers may be equipped to intervene and potentially delay the progression to AD.

Recent advancements in the understanding of cognitive aging and sensory impairments have underscored the critical role of early intervention in mitigating cognitive decline (Bateman et al., 2012). Studies have increasingly highlighted the interconnectedness between sensory deficits, particularly visual impairment, and cognitive functions such as memory and executive function (Yoshii et al., 2019). State-of-the-art neuroimaging techniques have allowed researchers to explore the neural substrates of these interactions, revealing that sensory and cognitive processing often share common neural pathways. These findings suggest that sensory decline, especially visual impairment, may exacerbate cognitive decline by overloading cognitive resources and altering brain function (Zhou, 2020). In the context of MCI, the role of visual function in both cognitive and daily functioning remains underexplored.

In this comprehensive cross-sectional study, we aim to investigate the role of visual function and ADL performance in cognitive function among individuals with MCI. We hypothesize that visual impairment will be associated with poor cognitive function and decreased performance on ADLs in individuals with MCI. Results will reveal the underlying mechanisms of cognitive decline in MCI and identify targets for intervention and support strategies.

## 2 Methods

### 2.1 Study design

We employed a multistage, stratified, cluster-sampling procedure to ensure representative sampling of the elderly population in Taicang City, Jiangsu Province. Taicang City has a total elderly population of 163,100 individuals aged over 60 years, constituting 30.6% of the city's population. In the initial stage of sampling, we randomly selected four districts from Taicang City, with two districts classified as economically developed (above the city's median GDP), and the two remaining districts classified as economically underdeveloped (below the city's median GDP).

**Figure 1:**
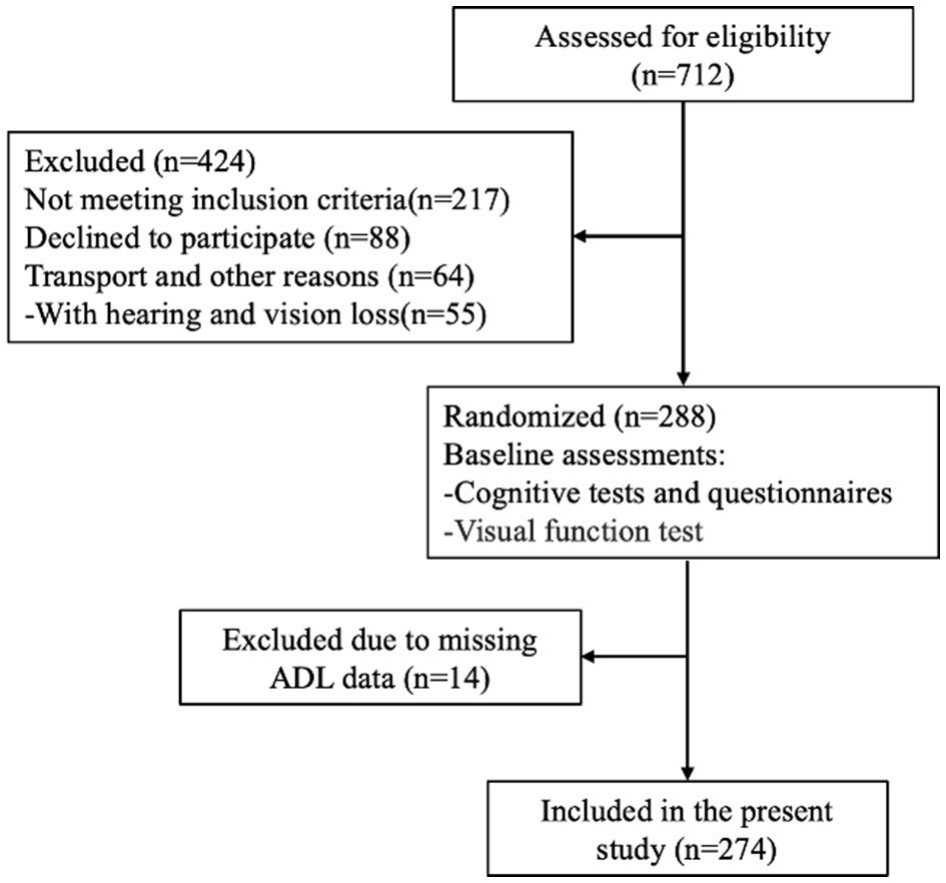
Flow chart of the study.

In the second stage of sampling, three communities were randomly chosen from each selected district. Each community typically comprises 500–700 households. Following community selection, we screened eligible elderly individuals based on predetermined recruitment criteria (Figure 1). The participants were required to have resided in Taicang for at least 1 year, while institutionalized individuals, such as those residing in nursing homes, were excluded from the study.

Twelve research sites participated in this study, and each contributed to comprehensive data collection efforts. This study was approved by the Scientific Research Ethics Committee of the Shanghai University of Sport (102772023RT200), and written informed consent was obtained from all participants prior to their inclusion in the study.

### 2.2 Participants

Over 700 elderly subjects aged 60 years or above were recruited from various communities in Taicang City. Recruitment efforts targeted community-dwelling older adults through local senior centers, community health clinics, and advertisements in community newsletters. The inclusion criteria were as follows: age of 65 years and above and diagnosis of MCI according to standardized criteria (Petersen, 2004). Additionally, participants were required to exhibit no significant visual or hearing impairment unrelated to aging, such as cataracts or macular degeneration, and to be capable of providing informed consent. self-reported cognitive decline was corroborated by healthcare providers when available. This approach aligns with the recommendations in some diagnostic guidelines for MCI when longitudinal data is unavailable.

Exclusion criteria were carefully delineated to ensure the integrity of the study sample. Individuals unable to cooperate with sensory and cognitive examinations due to conditions, such as deafness, blindness, or other sensory organ diseases, were excluded. Furthermore, participants suffering from major psychiatric disorders (e.g., depression, schizophrenia) or conditions that could confound cognitive function assessment (e.g., confusion, delirium) were excluded. Individuals experiencing cognitive impairment attributed to neuropsychiatric drug use were not eligible for participation. Finally, people who could not complete the cognitive test due to factors such as old age and low education (< 3 years) level were excluded. All participants were diagnosed by a physician who was a specialist in either geriatric medicine, psychiatry, or neurology. As a part of the diagnostic process, all subjects underwent ADL assessment, cognitive testing, and visual function examination. a trained Human Movement Sciences (HMS) research assistant tested the patients for the following additional inclusion criteria: Mini-Mental State Exam (MMSE) score of at least 9 to no more than 23 and the ability to perform the timed up-and-go test (Podsiadlo and Richardson, 2015), ADL score of < 26, indicating a reduced ability to perform daily tasks independently (Fuentes et al., 2020). In this study, 274 of the 288 participants (95.1%) had sufficient baseline data for variables of interest and were included.

### 2.3 Cognitive assessment

Cognitive assessments were performed in a quiet environment with no distractions. MoCA was used to assess global cognitive function (Yoshii et al., 2019). It includes tasks that assess various cognitive domains: visuospatial and executive functioning through a trail-making test (TMT), copying a cube (CC), and a clock-drawing test (CDT) by identifying three animals; memory with immediate and delayed recall of five words (M); attention via forward and backward digit span (FD and BD), a tapping test (TT), and serial 7s subtraction (S7S); language through sentence repetition (SR) and verbal fluency tasks (VFT); abstraction by explaining similarities between items; and orientation by providing the current date, month, year, day, place, and city. The total possible score on the MoCA is 30 points, with a score of 26 or above generally considered normal, while scores below 26 may indicate cognitive impairment, with lower scores suggesting significant impairment.

### 2.4 Assessment of ADL

ADL was assessed by the Alzheimer's Disease Co-operative Study-ADL (ADCS-ADL) scale (Galasko et al., 1997). The ADCS-ADL scale assesses 23 ADL items and is administered to the caregiver through an interview. The scale comprises two components, namely, basic ADL, which consists of six items, such as self-feeding, personal hygiene, and dressing; and instrumental ADL, which consists of 17 items, such as using telephone, reading books or magazines, managing finances, leisure activities, and household chores. The BADL score ranges from 0 to 22, and the IADL ranges from 0 to 56. BADL and IADL comprise the total ADL, with the range 0–78. High scores indicate less impairment.

### 2.5 Visual function assessment

Visual function was assessed utilizing an automated visual field test employing the Zeiss Visual Field Instrument HFA3 Model 860 to comprehensively evaluate the patient's visual field. The procedure entailed systematic analysis of the patient's visual field, wherein they fixated on a central point while targets were presented across various locations within their visual field. Responses were elicited through button presses or other means upon perception of the presented lights. Alongside determining the maximum visual field (Max VF) and minimum visual field (Min VF), the test outcomes were quantified through several key metrics. The VFI, which was expressed as a percentage, served as an indicator of overall visual field health, with 100% denoting normalcy and 0% indicating total blindness (Massof and Rubin, 2001). Mean Deviation (MD) represented the average deviation of all visual field points from the norm and reflected the extent of visual field loss; negative values signified defects, with larger negative values indicating greater severity (Rowe and Sarkies, 1998). Pattern Standard Deviation (PSD) gauged localized visual field damage by assessing inter-point variances, with elevated PSD values suggestive of irregularities, such as scotomasm, and typically correlating with localized visual field alterations. The examination included evaluation of visual acuity (VA), contributing to a comprehensive assessment of the patient's visual function (Warrian et al., 2010).

### 2.6 Statistical analysis

The unadjusted associations between cognitive function, as measured by the Montreal Cognitive Assessment (MoCA), and various measures of visual function (including Max VF, Min VF, VFI, MD, PSD, and VA, as well as total ADL, BADL, and IADL, were assessed using Pearson's correlation coefficient. In order to gain further insight into these relationships, multivariable linear regression models were constructed, taking into account age, sex, and educational levels. In these models, each measure of visual function and cognitive function was analyzed individually to determine the corresponding β-coefficients. A small number of patients had missing data, which were addressed through listwise deletion. All statistical analyses were conducted using SAS version 9.4 and R version 3.5.1. Statistical significance was set at *p* < 0.05.

## 3 Results

The baseline characteristics for the 274 participants are presented in Table 1. The average age was 69 years, with a mean MoCA score of 18.5, indicating MCI. A majority of participants were women (68.6%). The mean total ADL was 20.6, suggesting moderate impairment in daily living activities. Notable comorbidities included hypertension (41.6%) and diabetes (33.2%), indicating a higher burden of health conditions than typical older adult populations.

**Table 1:** Demographics.

**Characteristics**	**N 274 Mean (SD)**
Age, years (SD)	69 (5.1)
Gender (women), n (%)	188 (68.6%)
**Comorbidities, n (%)**	
Hypertension	91 (33.2)
Diabetes	53 (19.3)
Hypercholesterolemia	6 (2.2)
Apoplexia	3 (1.1)
Acute myocardial infarction	7 (2.6)
**Cognitive performance, mean (SD)**	
MoCA	18.5 (4.3)
Visuospatial and executive functioning	2.1 (1.4)
Naming	2.1 (0.9)
Memory	1.3 (1.5)
Attention	4.7 (1.3)
Language	1.5 (1.0)
Abstraction	1.5 (1.0)
Orientation	5.5 (0.8)
**Visual function mean (SD)**	
Max VF	136.4 (28.5)
Min VF	121.3 (32.9)
VFI	79.9 (16.9)
MD	−7.9 (6.5)
PSD	5.4 (2.2)
VA	4.7 (0.2)
**Activities of daily living, mean (SD)**	
ADL	20.6 (3.6)
BADL	8.0 (0)
IADL	12.4 (1.3)

MoCA, Montreal Cognitive Assessment; Max VF, Maximum Visual Field; Min VF, Minimum Visual Field; VFI, Visual Field Index; MD, Mean Deviation; PSD, Pattern Standard Deviation; VA, Visual Acuity; ADL, Activities of Daily Living; BADL, Basic Activities of Daily Living; IADL, Instrumental Activities of Daily Living.

Table 2 shows the β-coefficients from multivariable linear regression models adjusted for age and sex. Max VF was significantly positively correlated with the total MoCA score (r = 0.17, *p* < 0.05), but this correlation was not significant after adjusting for ADL (β = 0.12, *p* = 0.39). VFI showed a strong positive correlation with the total MoCA score (r = 0.61, *p* < 0.001), which weakened significantly after adjusting for ADL (β = 0.06, *p* = 0.23). Min VF had a negative correlation with the total MoCA score, which became positive after adjusting for ADL (β = 0.12, *p* = 0.04).

**Table 2:** Correlation and regression coefficients.

	**PCC**	**Regression coefficients (RC)**			**RC adjusted for ADL**		
	**r**	β	**95% CI**	***p*** **-value**	β	**95% CI**	***p*** **-value**
**MoCA**							
Max VF	0.17^**^	0.66	−0.01–0.03	0.47	0.12	0–0.04	0.39
Min VF	0.16^**^	−0.15	−0.02–0.02	0.87	0.12^*^	0–0.29	0.04
VFI	0.61^**^	0.58^*^	0.12–0.18	< 0.001	0.06	−0.01–0.28	0.23
MD	0.27^**^	−0.14	−0.20–0	0.06	0.07	−0.01–0.03	0.22
PSD	−0.23^**^	−0.10	−0.043–0	0.06	−0.1	−0.32–0.03	0.11
VA	0.21^**^	0.11	0.21–3.25	0.03	0.1	−0.23–3.07	0.09
ADL	−0.13^*^	0.04	−0.07–0.17	0.43			
BADL	0.02	0.01	−6.33–7.85	0.83			
IADL	−0.14^*^	−0.06	−0.54–0.12	0.23			
**Visuospatial and executive functioning**							
Max VF	0.13^*^	−0.01	−0.01–0	0.91	0.06	0–0.01	0.26
Min VF	0.17^**^	0.08	0–0.01	0.40	0.07	0–0.01	0.22
VFI	0.37^**^	0.35	0.01–0.04	< 0.001	0.06	0–0.01	0.29
MD	0.20^**^	−0.16	−0.07–0	0.08	0.07	0–0.01	0.21
PSD	−0.20^**^	−0.18	−0.19–0.03	0.01	−0.06	−0.09–0.02	0.31
VA	0.18^**^	0.12	0.01–1.12	0.04	0.02	−0.46–0.63	0.75
ADL	−0.09	−0.11	−0.09–0	0.06			
BADL	0.03	0.02	−2.08–3.10	0.70			
IADL	−0.08	0.02	−0.10–0.13	0.80			
**Naming**							
Max VF	0.02	−0.08	−0.01–0	0.49	0.11	0–0.01	0.1
Min VF	0.03	0.08	−0.004–0	0.43	0.16^*^	0.01–0.07	0.01
VFI	0.24^**^	0.24	0.01–0.02	0.00	0	0–0.01	0.99
MD	0.07	−0.09	−0.04–0.01	0.32	0.25	0–0.2	0.11
PSD	−0.07	−0.01	−0.06–0.01	0.91	−0.07	−0.6–0.01	0.29
VA	0.06	0.07	−0.17–0.62	0.27	0	−0.4–0.4	0.97
ADL	0.008	0.05	−0.02–0.05	0.41			
BADL	0.06	0.05	−1.11–2.63	0.43			
IADL	−0.005	−0.01	−0.09–0.08	0.91			
**Memory**							
Max VF	0.15^**^	−0.02	−0.01–0.01	0.87	0.09	0–0.1	0.17
Min VF	0.13^**^	−0.01	−0.01–0	0.96	0.1	0–0.1	0.09
VFI	0.33^**^	0.23	0.01–0.03	0.00	0.02	0–0.01	0.72
MD	0.19^**^	0.06	−0.03–0.05	0.48	0.19	0–0.03	0.22
PSD	−0.08	0.02	−0.08–0.10	0.82	−0.13^*^	−0.13- −0.1	0.03
VA	0.10	0.04	−0.41–0.81	0.51	0.05	−0.35–0.85	0.42
ADL	−0.14^**^	0.15	0.01–0.11	0.02			
BADL	−0.06	−0.06	−4.36–1.35	0.30			
IADL	−0.16^**^	−0.11	−0.26–0.01	0.08			
**Attention**							
Max VF	0.10	0.00	−0.01–0	0.97	0.09	0–0.01	0.18
Min VF	0.08	−0.05	−0.01–0	0.57	0.13^*^	0–0.01	0.03
VFI	0.40^**^	0.42	0.02–0.04	< 0.001	−0.01	−0.01–0.01	0.89
MD	0.20^**^	−0.18	−0.07–0	0.04	0.13	−0.1–0.02	0.4
PSD	−0.25^**^	−0.29	−0.26- −0.10	< 0.001	−0.01	−0.6–0.05	0.82
VA	0.15^**^	0.08	−0.13 - 0.90	0.14	0.03	−0.41–0.68	0.63
ADL	−0.08	0.04	−0.03–0.06	0.46			
BADL	0.06	0.04	−1.58–3.26	0.50			
IADL	−0.10	−0.03	−0.15–0.08	0.60			
**Language**							
Max VF	0.13^*^	0.14	0.00–0.01	0.18	0.11	0–0.01	0.11
Min VF	0.07	−0.06	−0.01–0	0.52	0.12^*^	0–0.01	0.05
VFI	0.35^**^	0.35	0.01–0.03	< 0.001	0.08	0–0.01	0.14
MD	0.16^**^	−0.18	−0.06–0	0.04	0.31^*^	0–0.03	0.05
PSD	−0.15^**^	−0.15	−0.12- −0.01	0.03	−0.05	−0.06–0.02	0.4
VA	0.15^**^	0.12	0.02–0.82	0.04	0.18^*^	0.2–1.0	0.003
ADL	−0.14^*^	0.02	−0.03–0.04	0.74			
BADL	0.03	0.02	−1.60–2.17	0.77			
IADL	−0.15^*^	−0.04	−0.12–0.06	0.55			
**Abstraction**							
Max VF	0.10	0.16	0.00–0.01	0.131	−0.03	−0.01–0	0.67
Min VF	0.08	−0.03	−0.01–0	0.786	−0.08	−0.01–0	0.23
VFI	0.27^**^	0.29	0.01–0.02	< 0.001	0.03	−0.01–0.01	0.58
MD	0.08	−0.12	−0.04–0.01	0.169	−0.11	−0.01–0.01	0.50
PSD	−0.05	0.01	−0.05–0.05	0.938	0.07	−0.01–0.05	0.26
VA	0.17^**^	0.10	−0.05–0.59	0.102	0.1	−0.62–0.57	0.11
ADL	−0.13^*^	−0.08	−0.05–0.01	0.195			
BADL	−0.07	−0.07	−2.50–0.54	0.203			
IADL	−0.12^*^	−0.02	−0.09–0.06	0.702			
**Orientation**							
Max VF	0.08	0.15	0.00–0.01	0.15	0.02	0–0.01	0.71
Min VF	0.07	−0.07	−0.01–0	0.47	−0.1	−0.01–0	0.86
VFI	0.26^**^	0.30	0.01–0.02	< 0.001	−0.01	−0.1–0.1	0.95
MD	0.12^*^	−0.10	−0.04–0.01	0.26	−0.29	−0.02–0	0.07
PSD	−0.15^*^	−0.09	−0.08–0.02	0.19	−0.12^*^	−0.07–0.01	0.05
VA	0.001	−0.10	−0.61–0.06	0.11	−0.09	−0.56–0.10	0.16
ADL	−0.009	0.03	−0.02–0.03	0.69			
BADL	0.03	0.03	−1.20–1.96	0.63			
IADL	0.01	−0.10	−0.13–0.02	0.13			

MoCA, Montreal Cognitive Assessment; Max VF, Maximum Visual Field; Min VF, Minimum Visual Field; VFI, Visual Field Index; MD, Mean Deviation; PSD, Pattern Standard Deviation; VA, Visual Acuity; ADL, Activities of Daily Living; BADL, Basic Activities of Daily Living; IADL, Instrumental Activities of Daily Living. The symbols ^*^ and ^**^ represent statistical significance: ^*^ indicates *p* < 0.05 and ^**^ indicates *p* < 0.01.

VFI was significantly positively correlated with memory (r = 0.33, *p* < 0.001) and attention (r = 0.40, *p* < 0.001), but these correlations were not significant after adjusting for ADL (memory: β = 0.02, *p* = 0.72; attention: β = −0.01, *p* = 0.89). Max VF was significantly positively correlated with language (r = 0.13, *p* < 0.05), and this correlation remained significant after adjusting for ADL (β = 0.11, *p* = 0.11).

Regression analysis indicated that ADL moderated the relationship between visual function and cognitive performance. VFI's strong positive correlation with the total MoCA score before adjustment (β = 0.58, *p* < 0.001) weakened after adjustment (β = 0.06, *p* = 0.23). Min VF's negative correlation with memory became positive after ADL adjustment (β = 0.10, *p* = 0.09). Overall ADL was negatively correlated with memory (r = −0.14, *p* < 0.05), indicating that lower ADL capabilities are associated with poorer memory performance.

## 4 Discussion

This study underscores the intricate relationship between visual function and cognitive performance in older adults, highlighting the critical moderating role of ADL. The observed correlations between visual function indices (Max VF, VFI, Min VF) and cognitive performance (MoCA score) align with previous research showing that visual impairments can detrimentally affect cognitive abilities (Zanto et al., 2011; Kalkstein et al., 2011). The positive correlation between Max VF and cognitive performance, especially in language abilities, suggests broader visual fields contribute to improved cognition (Clemmensen et al., 2020; Lindenberger et al., 2008). However, the attenuation of these correlations after adjusting for ADL emphasizes that daily functional activities significantly mediate the effect of visual function on cognitive performance (Raimo et al., 2024; Clemmensen et al., 2020; Tulliani et al., 2022). This finding suggests that interventions should not only focus on visual rehabilitation but also on enhancing ADL capabilities.

The initialnegative correlation between Min VF and cognitive performance, which became positive after adjusting for ADL, indicates a nuanced interaction (Wolf et al., 2021; Eckstein et al., 2017). While a reduced visual field might initially hinder cognitive abilities, the context of daily activities can shift this effect. This may be due to compensatory mechanisms where individuals develop alternative strategies to maintain cognitive performance despite visual impairments (Buele et al., 2023; Jia et al., 2019). Additionally, the reversal of the Min VF and cognitive performance relationship post-ADL adjustment is a novel finding that suggests a complex interaction requiring further investigation (Guo et al., 2021). Enhancing ADL capabilities could buffer the effects of visual impairments on cognitive health, offering a more detailed understanding of how visual function impacts different cognitive processes.

Healthcare professionals should adopt a holistic approach that integrates assessments of visual function, cognitive performance, and ADL to develop effective intervention strategies. Rehabilitation programs should enhance visual fields and ADL skills, recognizing their synergistic potential in improving cognitive outcomes (Wolf et al., 2021; Eckstein et al., 2017). This strategy is particularly relevant for memory and language domains, where the interplay between visual function and ADL is most pronounced (Jia et al., 2019). Future research should use longitudinal designs to track changes over time and better understand how visual impairments and ADL interact to influence cognitive health.

This study provides valuable insights into the relationship between sensory impairments, cognitive function, and daily living activities in older adults; however, several limitations should be considered. The sample was relatively homogenous, limiting the generalizability of the findings, and the cross-sectional design prevents establishing causal relationships between sensory deficits and cognitive decline. Future research should incorporate longitudinal designs to explore causality and include more diverse populations to improve generalizability. Additionally, the reliance on self-reported IADL performance may introduce bias, and future studies should utilize objective, performance-based assessments. While this study focused on cognitive and sensory impairments, other factors such as mental health and social engagement should also be considered in future research. Exploring interventions that target both sensory and cognitive domains, particularly those integrating technologies like wearable devices or cognitive training apps, could offer promising avenues for intervention. Finally, investigating the neurobiological mechanisms linking sensory impairments and cognitive decline, including biomarkers and neuroimaging, could further enhance our understanding and inform preventive strategies. Addressing these limitations and exploring these future directions will be crucial for advancing the field and developing effective interventions for older adults at risk of cognitive decline.

## 5 Conclusion

This study elucidates that visual function significantly influences cognitive performance, with ADL as a crucial moderating factor. Enhanced visual function is correlated with superior cognitive performance, and the moderating effect of ADL suggests that improvements in ADL capabilities could positively affect cognitive health. These findings underscore the importance of comprehensive assessments and interventions to enhance visual function and ADL capacities and bolster cognitive health in the elderly population. Future research should focus on developing and evaluating interventions designed to simultaneously improve ADL and visual function to optimize cognitive outcomes in older adults.

## Data Availability

The original contributions presented in the study are included in the article/supplementary material, further inquiries can be directed to the corresponding author.
